# Are Diatoms “Green” Aluminosilicate Synthesis Microreactors for Future Catalyst Production?

**DOI:** 10.3390/molecules22122232

**Published:** 2017-12-16

**Authors:** Lydia Köhler, Susanne Machill, Anja Werner, Carolin Selzer, Stefan Kaskel, Eike Brunner

**Affiliations:** 1 Chair of Bioanalytical Chemistry, Faculty of Chemistry and Food Chemistry, TU Dresden, 01062 Dresden, Germany; lydia.koehler@tu-dresden.de (L.K.); susanne.machill@tu-dresden.de (S.M.); 2 Institute of Inorganic Chemistry, Faculty of Chemistry and Food Chemistry, TU Dresden, 01062 Dresden, Germany; anja.werner@tu-dresden.de (A.W.); carolin.selzer@tu-dresden.de (C.S.); stefan.kaskel@tu-dresden.de (S.K.)

**Keywords:** diatoms, biosilica, aluminosilicate, catalysis

## Abstract

Diatom biosilica may offer an interesting perspective in the search for sustainable solutions meeting the high demand for heterogeneous catalysts. Diatomaceous earth (diatomite), i.e., fossilized diatoms, is already used as adsorbent and carrier material. While diatomite is abundant and inexpensive, freshly harvested and cleaned diatom cell walls have other advantages, with respect to purity and uniformity. The present paper demonstrates an approach to modify diatoms both in vivo and in vitro to produce a porous aluminosilicate that is serving as a potential source for sustainable catalyst production. The obtained material was characterized at various processing stages with respect to morphology, elemental composition, surface area, and acidity. The cell walls appeared normal without morphological changes, while their aluminum content was raised from the molar ratio *n*(Al):*n*(Si) 1:600 up to 1:50. A specific surface area of 55 m^2^/g was measured. The acidity of the material increased from 149 to 320 µmol NH_3_/g by ion exchange, as determined by NH_3_ TPD. Finally, the biosilica was examined by an acid catalyzed test reaction, the alkylation of benzene. While the cleaned cell walls did not catalyze the reaction at all, and the ion exchanged material was catalytically active. This demonstrates that modified biosilica does indeed has potential as a basis for future catalytically active materials.

## 1. Introduction

Currently, heterogeneous catalysts play an essential role in chemical industry. Especially zeolites are widely used as highly active and selective catalyst materials in various reactions, such as catalytic cracking, alkylation, and isomerization [1]. These crystalline aluminosilicates are mostly produced by hydrothermal synthesis on an industrial scale [2]. Some of their varieties also occur as minerals in the earth’s crust [3].

The present study describes a bioinspired approach towards functional aluminosilicate materials from a natural plant source, namely diatoms. Their “green” production is environmentally friendly and inexpensive. Diatoms are unicellular, mostly phototrophic algae inhabiting oceans, lakes, and ponds, as well as various soils and even rocks. With their uniquely structured and porous siliceous cell walls, the so-called frustules, diatoms stand out from related algae. These species-specific frustules serve as mechanical support, as well as mechanical protection against herbivores, and consist mainly of amorphous silica [4,5,6,7].

So far, the cell walls of diatoms are mostly used in the form of diatomite in numerous applications like filtration, abrasion, and chromatography [8]. As catalyst support, frustules of cultured diatoms have shown to be potentially more effective than diatomite [9]. Diatomite consists of fossilized diatoms in a mixture with a considerable amount of other minerals. Hence, the material is chemically impure and the aged silica fraction differs widely from that of the original frustules with a higher silanol (Si-OH) to siloxane (Si-O-Si) ratio [10]. Silanol groups are supposed to be partially deprotonated in the relevant neutral pH range [11]. Thus, they are weakly acidic—a positive aspect for acid catalysis. Cultured diatoms also offer the advantages of both a higher percentage of whole cells and the absence of other diatom species. Therefore, biosilica from cultured and freshly harvested diatoms was studied here.

This material may, however, be further improved. Two basic approaches exist to functionalize diatoms: (i) in vivo by genetic engineering or by changing growth parameters during the cultivation; (ii) in vitro by chemical or physical treatment after cell death. The latter is commonly used for diverse applications. For instance, iron nanoparticles that were treated with dopamine were coupled to frustules in order to obtain magnetic silica particles [12]. Another example is diatomite that was modified with aluminum and successfully tested in sewage treatment [13].

In vivo treatment of diatoms with additives is possible when the applied chemicals are incorporated into the algae. Such chemicals may be taken up by the diatoms and attached to the biosilica. Adopting this method, frustules modified with titanium, which were photocatalytically active [14], with thiols [15] and with fluorescence markers [16] were reported. The latter resulted in homogeneously glowing valves.

Apart from silica, diatom frustules naturally contain a multitude of organic compounds, like polypeptides [17], polyamines [18], and polysaccharides [19], as well as elements like iron [20], zinc [20], germanium [21], calcium [22], or aluminum [23]. The present study highlights the potential of aluminum-enriched biosilica. Aluminum is not an essential nutrient for plants, but it is known to be incorporated into diatom cell walls. For this reason, algal growth has a biogeochemical impact upon the global aluminum cycle. Algal blooms cause a decline of the aluminum concentration in waters, as could be observed in the North Sea [24] and in the Chinese Sea [25].

The aluminum enrichment in diatom cell walls may result from detoxification processes. Aluminum concentrations of a few µg/L can already be harmful to algae, leading to growth inhibition and cell death, which potentially eliminates species in waters with high aluminum levels [26]. This toxicity may result from the interference of aluminum with photosynthesis, declining the electron transport rates in thylakoid membranes [27]. Subsequently, more reactive oxygen species are produced. Glycolysis and pentose phosphate pathway are up-regulated in order to cover the cell energy demand, minimizing the toxic effects of aluminum [27]. Additionally, aluminum impacts the bioavailability of phosphate, which is essential for diatoms [28]. It is therefore speculated that the cell removes the toxic aluminum by binding it to the siliceous cell walls.

Note that the extracellular matrix of diatoms consists, to a large extent, of (poly)saccharides [29]. This kind of organic coating is known to bind metal ions [30]. The specific influence of the organic coating upon Al uptake is not yet known and may be subject of future investigations.

Another reason for the enrichment of aluminum in diatom frustules may be a stabilizing effect of aluminum upon the silica. In vitro experiments revealed that even small amounts of attached aluminum decelerate the hydrolysis of amorphous silicate [31]. This effect occurred in raw diatom frustules, as well as in whole diatom cells post-mortem when they were exposed to an aluminum enriched solution [32,33]. Aluminum was incorporated into the silica, forming tetrahedral AlO_4_^−^. Adsorbed aluminum accumulated after a longer time period, i.e., hours or days of exposure [33]. These observations are in accordance with studies on silica gel precipitates, showing tetrahedral aluminum with chemical bonds to the silica, while octahedral aluminum is adsorbed at the silica surface [34].

The influence of culturing conditions on the aluminum uptake is complex. For instance, aluminum may react with other substances in the growth medium, e.g., with phosphorus [28] or dissolved organic matter [35], forming coordination compounds. On the other hand, aluminum can apparently increase the uptake of organic phosphorus under certain conditions [36]. Furthermore, silicon was reported to alleviate aluminum toxicity by co-accumulating with aluminum at the extracellular matrix of the diatom [37].

Naturally, the aluminum concentration in diatom frustules is rather small. For example, the molar Al:Si ratio in cultured *Stephanopyxis turris* has been determined as 1:1800 [23]. In former studies, the aluminum content could be increased up to 1:15 by increasing the aluminum concentration in the growth medium [23]. This Al:Si ratio is in the range typically found in zeolites, such as ZSM-5. In comparison to zeolite materials, the production of biosilica has economic and ecological advantages because it takes place under ambient conditions and near neutral pH. The question is, therefore, whether the biological material could in the future be used as adsorbent and catalyst in a similar way as zeolites.

The centric diatom *Thalassiosira pseudonana* (*T.p.*) was used in the present study as a well-established model organism [38]. This species is fast growing (1.4 d^−1^ at 20 °C and 12 h/12 h light/dark cycle [39]) and is resistant to environmental stress, which is underlined by its almost worldwide distribution. *T.p.* occurs in oceans, rivers, and lakes. Therefore, it can adapt to more diverse conditions—including chemicals, environmental conditions, and perturbations—than diatoms from stable environments [40]. It has been assumed that *T.p.* has developed especially effective detoxification mechanisms [41]. Accordingly, this species grows in a pH range from 6.8 to 8.4 at stable multiplication rates [42]. It tolerates salinities from 0.5–37‰ [43] as well as temperatures from 4 °C to 25 °C [44].

The present study aims at increasing the aluminum concentration in diatom frustules for catalytic applications and demonstrates two types of biosilica modification procedures. The obtained material was characterized with regard to different chemical and physical aspects: elemental composition, morphology, pore surface area, acidity, and catalytic activity.

## 2. Results and Discussion

### 2.1. Diatom Cultivation and Microscopic Characterization

Preliminary tests did not reveal any detectable toxic effects of aluminum upon *T.p.* within the solubility range of AlCl_3_, except for a delayed cell growth. The aluminum concentration that was chosen for further cultivation was 2.7 mg/L, i.e., 100 times the concentration that is in standard artificial seawater (ASW). This is close to the solubility limit.

During cultivation, the vitality of diatom cells was monitored in vivo by light microscopy. The cells appeared normal without visible deformations. After approximately four weeks, cells were harvested and cleaned. *T.p.* has a sufficiently silicified frustule that can withstand most non-alkaline chemicals and thermal treatment.

Subsequent to cleaning and the calcination of the siliceous cell walls, their structure was examined with SEM. The structure is relevant to the porosity and specific surface area, and hence to later applications; especially a loss of porosity would be undesirable.

**Figure 1:**
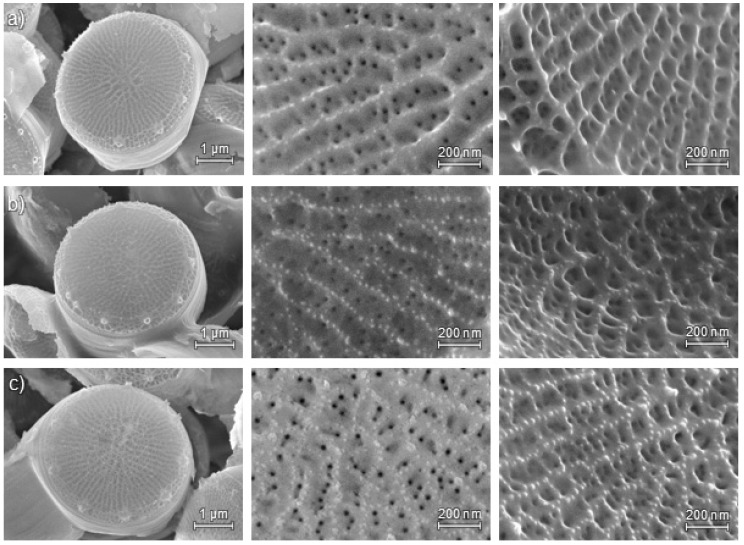
SEM images of calcined *T.p.* frustules; left: whole valve, center: detailed frontal view, right: detailed view with tilted valve face; (**a**) reference sample, (**b**) cultured under elevated aluminum levels; (**c**) cultured under elevated aluminum levels, after ion exchange.

Note that the morphology of *T.p.* varies to a certain degree even under common environmental conditions. Diatom frustules are built similar to a petri dish, consisting of two valves and adjacent girdle bands. Depending on the species, the cell walls contain various structural elements, like processes, hierarchical pores, and ridges that are arranged in particular patterns. The *striae* (rows of pores) and *costae* (ridges) form the distinct structure of the valve face of *T.p.* Regarding the pore size, large mesopores and macropores are discernable via SEM. In the examined *T.p.* cultures, cell size, cell shape, form and number of *portulae* (special pores, partly with processes), and girdle bands were apparently unperturbed by aluminum enrichment (Figure 1).

### 2.2. Chemical Structure and Elemental Composition

Silica consists of silicon atoms, each bound to four oxygen atoms. These SiO_4_ tetrahedra build an irregular network. When foreign atoms are incorporated, they can be intercalated, thus filling gap sites in the structure. Furthermore, they can also be directly inserted into the silica network structure. The latter commonly occurs when foreign atoms are chemically similar to silicon. Therefore, elements like germanium [21] and aluminum [45] can replace silicon atoms in the framework. In the case of aluminum, a negative framework charge as in zeolites is created.

**Figure 2:**
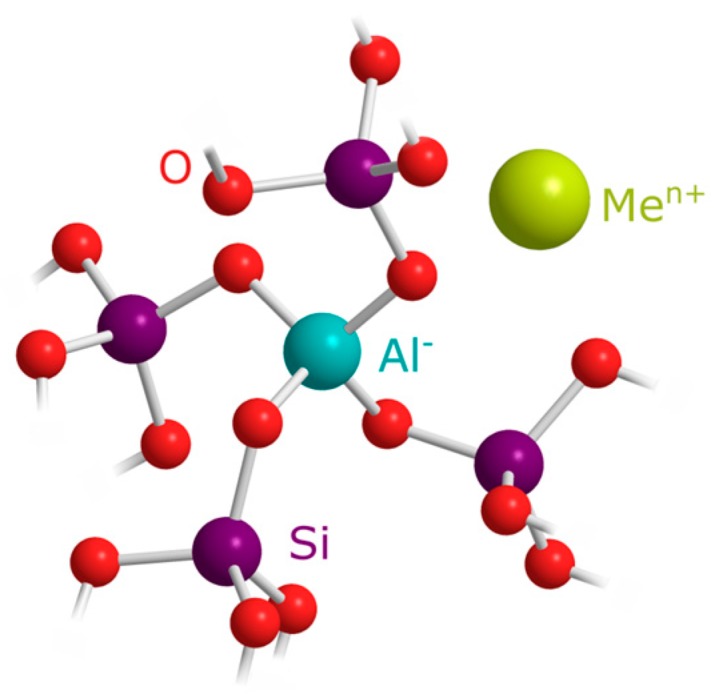
Structure of aluminum in the silica framework with Me^n+^ as counter ion; model adapted from [47].

This charge can be counterbalanced by positively charged metal ions (Me^n+^), which are present in the growth medium, as depicted in Figure 2. The artificial seawater used in this study closely resembles natural seawater, containing a high concentration of sodium, but also calcium, magnesium, and potassium. Former studies reported several alkali and alkaline earth metals in diatom cell walls [46]. Moreover, Gehlen et al. elucidated the structure of biogenic silica that is enriched with aluminum. A molar Ca:Al ratio of 1:2 could be observed [45,47]. In this ratio, Ca^2+^ would exactly neutralize the charge of AlO_4_^−^.

Theoretically, aluminum could also be bound to the diatoms’ extracellular matrix or be enriched in their organic cellular compartments. For instance, a slow adsorption within the course of weeks and months, as well as a more rapid, stronger binding of aluminum have been reported [33]. These effects are, however, negligible under the conditions and methods that are employed in the present paper, as was verified in preceding experiments (described in Section 3.2).

Frustules enriched with aluminum are not catalytically active after harvesting and cleaning. However, aluminum enriched biosilica might become catalytically active after an ion exchange replacing the metal ions, such as Na^+^ and Ca^2+^, by ammonium ions. Ammonia removal will then result in the formation of so-called bridging OH groups at Si-O-Al units, i.e., Brønsted acid sites.

**Figure 3:**
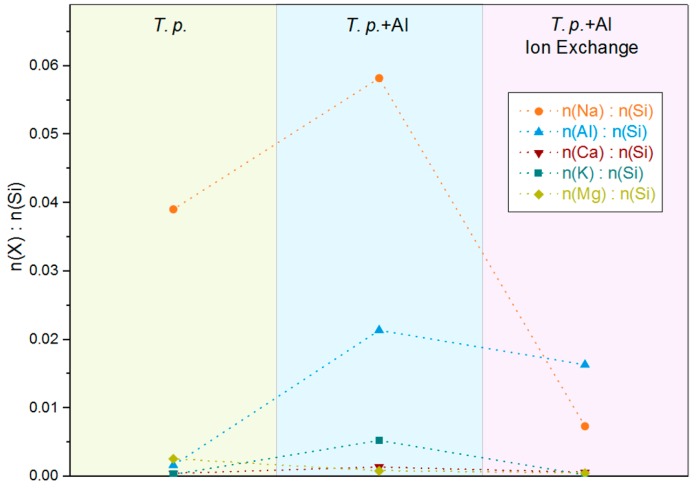
Molar ratios of several elements to Si in the biosilica of *T.p.* cultured in standard ASW (*T.p.* Reference) as well as *T.p.* cultured in artificial seawater (ASW) enriched with aluminum, calcined only (*T.p.* + Al) and with additional ion exchange (*T.p.* + Al Ion Exchange); dashed lines to connect ratios of the same elements.

The determined molar element to silicon ratios for several foreign elements that are found in the biosilica are listed in Table 1 and visualized in Figure 3. The aluminum content of the frustules cultured under elevated aluminum levels considerably increased by more than one order of magnitude from an Al:Si ratio of about 1:600 to 1:50. Thus, the culture conditions that were previously tested with *Stephanopyxis turris* were successfully transferred to *T.p*. It is advantageous that *T.p.* produces the desired material substantially faster, i.e., with a higher growth rate than *Stephanopyxis turris*. Additionally, the high tolerance of *T.p.* probably allows even higher aluminum concentrations if the aluminum solubility can be further enhanced, e.g., by complexing agents.

Along with the aluminum concentration, the relative amount of almost all of the investigated cations increased, except for magnesium. This is expected if an isomorphous substitution of silicon by aluminum in the silica framework takes place. The increasingly negative charge of the framework must then be compensated by an increasing number of alkali and alkaline earth metal cations. These results are in accordance with the model of Gehlen et al. [45,47]. However, Gehlen et al. stressed the role of calcium for the diatoms *Thalassiosira nordenskjoeldii* and *Lauderia annulata*. In the present paper dealing with *T.p.*, sodium was identified as the main counterion after cell wall extraction and cleaning. In the samples that are analyzed here, the increase of the molar Na:Si ratio from 1:26 to 1:17 correlates well with the increase of the Al:Si ratio. When considering the composition of seawater, the importance of sodium seems obvious. In comparison, the concentrations of potassium and calcium are low. Nevertheless, an increase from 1:3250 to 1:189 and from 1:2220 to 1:715, respectively, is observed for potassium and calcium.

The ion exchange with ammonium chloride and the subsequent calcination reduces the relative amount of the analyzed alkali and alkaline earth metals because they are replaced by protons. Sodium shows a decrease of the molar Na:Si ratio from 1:17 to 1:136 after ion exchange. The K:Si ratio is reduced from 1:189 to 1:4190. To confirm that the ion exchange procedure indeed results in a higher proton concentration causing a higher acidity, the material was furthermore analyzed by NH_3_ TPD (see below).

### 2.3. Nitrogen Physisorption

The surface area and porosity are essential properties for the catalytic activity of a material. SEM revealed large mesopores and macropores in the biosilica. Macropores offer the advantage of good accessibility, providing unlimited mass transfer to internal adsorption sites. This is particularly important for large reactant molecules. Furthermore, product molecules are released more easily because blocking of macropores is less probable [48]. However, the relatively low specific surface area of a macroporous material is a clear disadvantage since it is associated with a relatively low adsorption capacity, which negatively affects the catalytic performance. Reactants bind rather weakly to pores having a distinctly larger diameter than the kinetic diameter of the reactant molecules.

The physisorption of nitrogen at −196 °C is particularly suitable for the analysis of micropores and mesopores, which are most relevant to the catalytic activity. Thus, N_2_ physisorption was used to estimate the surface area of the aluminum enriched biosilica material. The BET method reveals a specific surface area of 55 m^2^/g. As macropores are not filled under the applied conditions, the surface area may be slightly underestimated. The calculated surface area is comparable with the values that are typically found for other biominerals [49]. When comparing *T.p.* enriched with aluminum and grown without aluminum addition, there is little difference regarding the pore volumes (*T.p.* + Al: 0.4 cm^3^/g, standard *T.p.*: 0.3 cm^3^/g [9]).

**Figure 4:**
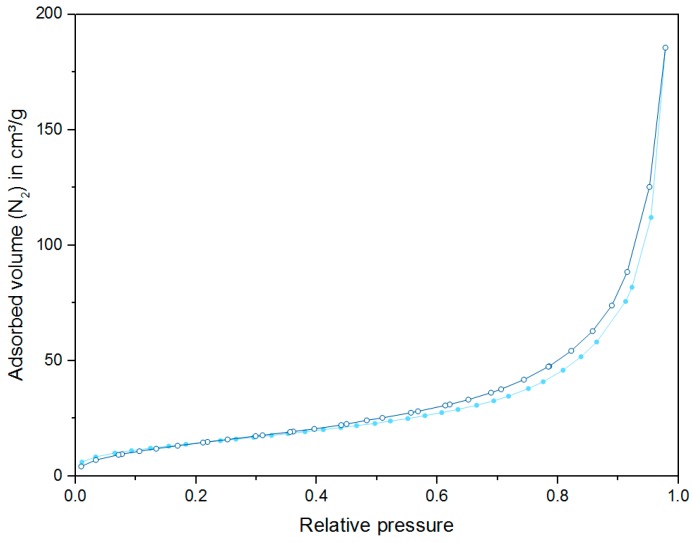
Nitrogen adsorption (light blue, solid circles) and desorption (dark blue, open circles) isotherm of *T.p.* enriched with aluminum.

The adsorption isotherm depicted in Figure 4 exhibits a rather gradual, mostly linear slope up to a relative pressure of 0.8. The steep N_2_ uptake curve in the high relative pressure range is attributed to unrestricted multilayer adsorption as it is observed in large mesopores and macropores. The hysteresis loop located in the multilayer range is indicative for the presence of mesopores [50].

### 2.4. NH_3_ TPD

**Figure 5:**
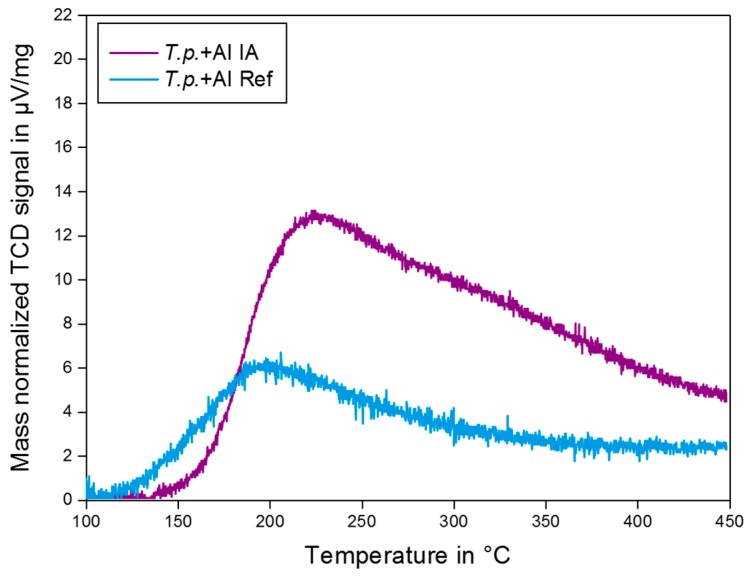
TPD profiles of biosilica enriched with aluminum, calcined only (blue) and with additional ion exchange (violet).

Temperature programmed ammonia desorption offers valuable information about the strength and concentration of acidic sites. When ammonia is adsorbed on an acid surface, the strength of the acid adsorption site determines the ammonia binding energy. Strongly bound ammonia molecules desorb from the surface at higher temperatures than weakly bound molecules. Hence, the peak temperature in a TPD profile provides information on the strength of acid sites. The area that is underneath the curves reflects the number of sites. The measured ammonia desorption curves of biosilica before and after ion exchange are shown in Figure 5.

A total acid site concentration of 149 µmol NH_3_/g for the calcined Al enriched biosilica and 320 µmol NH_3_/g for the biosilica after ion exchange was calculated from the area under the curves. Evidently, the ion exchange was successful, as indicated by the increase of the acid site concentration by more than a factor of 2. Both of the TPD profiles show a peak at relatively low temperatures of about 200–210 °C attributed to ammonia desorbing from weaker acid sites, potentially Lewis acids originating from extra-framework aluminum species and/or free metal cations.

TPD measurements with additional water vapor treatment of ammonia loaded zeolites samples, performed by Katada et al. revealed that the low-temperature peak was caused by ammonia adsorbed on extra-framework metal cations owing to the electrostatic interaction of the N-H bond with the cation [51]. According to Katada et al. the intensity of the low-temperature peak is not relevant to the catalytic activity in acid-catalyzed reactions.

**Figure 6:**
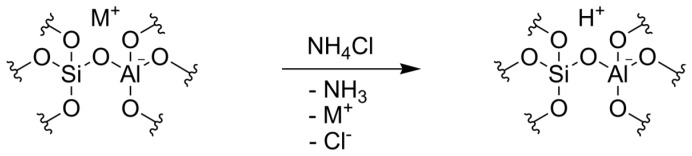
Ion exchange with ammonium chloride, generating Brønsted acid sites in the Al enriched biosilica after ammonia removal.

In contrast to the TPD profile of the calcined biosilica, the desorption curve of the ion exchanged silica additionally exhibits a broad, overlapping peak around 350 °C, indicating the presence of stronger acid sites. Presumably, this peak is attributed to acidic protons, i.e., Brønsted acid sites, as introduced by the ion exchange (Figure 6).

Such acidic sites would be active in acid-catalyzed reactions. One should keep in mind that the TPD method cannot discriminate the nature of acid sites. However, the results of the TPD experiments clearly demonstrate the beneficial effect of the ion exchange on the acidity of the biosilica material that is caused by the exchange of the extra-framework metal cations with acidic protons.

### 2.5. Catalytic Tests

**Figure 7:**
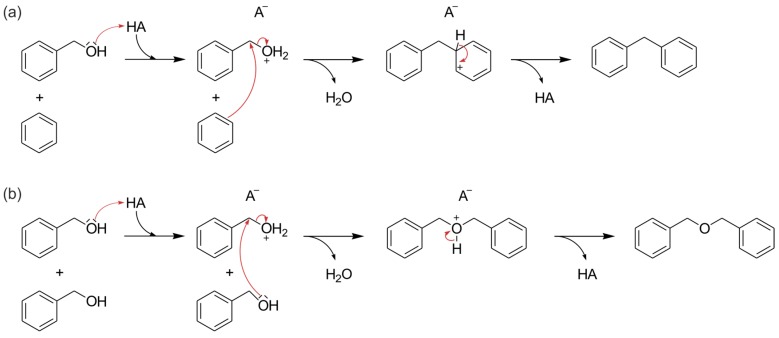
Reaction mechanism of the Brønsted acid (HA) catalyzed formation of (**a**) diphenylmethane and (**b**) dibenzyl ether [53,54].

The catalytic activity of the biosilica was evaluated in the liquid-phase alkylation of benzene with benzyl alcohol. Industrially, this reaction is usually catalyzed by zeolites [52]. It necessarily requires the presence of a catalyst, i.e., does not start under the chosen conditions without a suitable acid. Both Brønsted and Lewis acid sites are catalytically active in the alkylation. In Figure 7, the reaction mechanism is exemplarily shown for the Brønsted acid catalyzed route. The bond of the hydroxyl group to the benzyl group is weakened by the addition of the catalytic Brønsted proton. Thus, diphenylmethane is formed via an aromatic addition alongside other possible products, such as dibenzyl ether [53,54].

**Figure 8:**
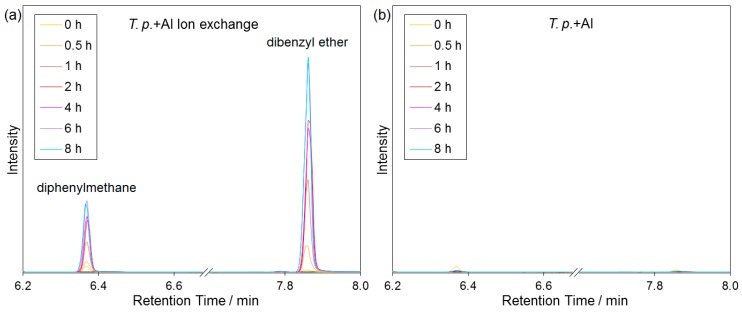
Chromatograms of the reaction mixture (section of the product peaks); (**a**) with Al enriched and ion exchanged biosilica (*T.p.* + Al Ion exchange) and (**b**) with Al enriched biosilica without ion exchange (*T.p.* + Al); chromatograms normalized to the same amount of internal standard; and, selected ion traces (6.2–6.87 min: *m*/*z* 167, 6.87–8.0 min: *m*/*z* 92).

The test reaction was monitored by Gas Chromatography Coupled to Mass Spectrometry (GC-MS). In the test with aluminum enriched biosilica without ion exchange after calcination, none of the expected products occurred, except for small impurities, which are already present in the educt mixture (Figure 8b). Using ion exchanged biosilica, the formation of both diphenylmethane (ca. 6.4 min) and dibenzyl ether (ca. 7.9 min) was observed in the chromatogram (Figure 8a). This shows the catalytic activity of the Al enriched biosilica after ion exchange and ammonia removal.

Note that the observed catalytic activity is not yet comparable with zeolites. The obtained total product yield (related to benzyl alcohol) was only about 3%. This is not surprising since the number of acidic sites and their accessibility play a crucial role for the catalytic activity. Since the pores of the biosilica are rather large as shown by nitrogen physisorption measurements and SEM, the accessibility of the active sites is assumed to be unrestricted. Therefore, the drawback of the material may be the relatively low surface area and the amorphous nature of the biosilica. This results in a low adsorption capacity.

The catalytic performance may benefit from further chemical modifications of the cleaned frustules aiming to increase the adsorption capacity. One approach is to enhance the specific surface through micro- and mesopores. For instance, smaller mesopores could be introduced by mild desilication with bases like tetraethylammonium hydroxide, as it is known from zeolite science [55]. In this way, the surface area could be increased, while additionally elevating the aluminum content, and thus the acid site concentration. A higher number of acid sites is expected to provide an enhanced catalytic activity. This could also be achieved by increasing the relative aluminum concentration in vivo. Provided that the chosen diatom species tolerates these changes, several parameters could be adjusted during cultivation, like additional complexing agents or a different pH. More alkaline conditions could increase the bioavailability of aluminum since its minimal solubility is at approximately pH 6.5 [56]. Furthermore, one might also consider other diatom species for analogous experiments as shown here. Species with an intrinsically high degree of silicification seem to be particularly promising in this context [57,58].

A further interesting approach is to change the biosilica structure. In principle, amorphous silica can be transferred in a crystalline material [59]. There are two basic methods for this “zeolitization”: Previously synthesized crystalline silica nanoparticles can be fixed on the surface of the amorphous silica. They serve as crystallization seed in the subsequent hydrothermal treatment [60]. Another option is the partial dissolution of the amorphous biosilica. The dissolved material can recrystallize at the surface of the residual parts [61].

Thus, there are a lot of different ways to improve the biosilica from diatoms. In the long term perspective, high production volumes of algae that are grown under natural light show great promise in many respects. While binding CO_2_, algae produce lipids like triacylglycerids and sterols, which can be industrially used in the production of important goods like food, animal feed, pharmaceuticals, fertilizers, and biofuel [62,63]. Diatom biosilica could thus be a beneficial co-product with application potential as catalyst material as is demonstrated in the present work.

## 3. Materials and Methods

Some of the basic applied methods were described in former articles of this research group: cultivation, cleaning, Inductively Coupled Plasma Optical Emission Spectroscopy (ICP-OES), and SEM [23], as well as TPD [64].

### 3.1. Cultivation

*T.p.* (strain SAG 1020-1b, obtained from the Universität Regensburg, Regensburg, Germany) was axenically grown in 20 L carboys under a 12 h/12 h light/dark regime with about 1000 Lux in RUMED 1301 (Rubarth Apparate, Laatzen, Germany) and PLG400 (Lintek, Eutin, Germany) thermostats at 21 °C. This regime was chosen in order to be comparable with the conditions that were set in previous experiments [23]. The effect of light/dark cycle on the incorporation of elements varies depending on the substance. While the carbon and nitrogen uptake, for example, are increased with higher irradiance [65], the effect of light on aluminum incorporation is yet to be elucidated. An influence of irradiation on aluminum is conceivable, since aluminum toxicity is connected to photosynthesis [27] and the photosynthesis rate can be higher in cells under longer irradiance in the light/dark cycle [66]. The culture medium was ASW, with a composition according to the North East Pacific Culture Collection [67] (see supporting information). The standard concentration of aluminum in this water was determined as 0.027 mg/L, which was caused by impurities of the applied chemicals. The hundredfold concentration, i.e., 2.7 mg/L or 106 µmol/L, was applied for aluminum enrichment after initial toxicity evaluation in culture plates with 2.5 mL volume per well. To increase solubility, aluminum was added in form of dissolved aluminum chloride complexed by bis-tris methane (buffer quality, AppliChem, Darmstadt, Germany).

Independent of Al, the pH of the culture medium rises during diatom growth due to the algae’s metabolism. Assuming that only Al^3+^ is incorporated, dissociated ligands, like OH^−^ [68], could further increase the pH of the surrounding medium. Consequently, the rising pH was adjusted as necessary to pH 8 with hydrochloric acid. After about four weeks, the silicon concentration was depleted to under 5 µmol/L, as colorimetrically determined by the molybdenum blue method according to Iler [69]. Cultures were harvested with a Heraeus Megafuge 40 centrifuge (4200 rpm; Heraeus Holding, Hanau, Germany). The obtained algae pellets were washed five times with ultrapure water, and were frozen at −65 °C afterwards.

### 3.2. Cleaning of the Frustules

Before cleaning, the algae pellets resulting from nine different batches were thoroughly mixed. The material was then treated with lysis buffer, a mixture of 0.069 mol/L SDS (Merck, Darmstadt, Germany), and 0.10 mol/L EDTA (Grüssing, Filsum, Germany) with pH 8. This mixture was heated to 95 °C for 10 min, centrifuged, and the supernatant was discarded. The lysis step was repeated until the supernatant appeared mostly colorless. Subsequently, the pellets were washed five times with ultrapure water.

Remaining organic compounds were removed via calcination in a muffle furnace under static air conditions. After heating to 550 °C with a rate of 2 °C/min, the temperature was kept for 10 h.

In order to ensure that this material only contains tightly silica-attached Al species, we performed the following experiment. After harvesting and killing the cells, one half of a batch was washed and stored in ultrapure water, while the other half was left in Al-containing ASW. After one week at room temperature, biosilica from both of the samples was extracted as described above and analyzed via ICP-OES. There was no significant difference in the molar Al:Si ratios. That means, potentially adsorbed Al from the ASW is just weakly bound to the surface or located in organic cellular components and, consequently, removed during the applied lysis and washing steps.

### 3.3. Ion Exchange

First, the calcined biosilica was stirred in an aqueous solution of 0.8 mol/L ammonium chloride at 80 °C for 8 h. The solid was then washed five times in ultrapure water and dried afterwards. Subsequently, the material was heated in a muffle furnace with 5 °C/min to 550 °C to drive out ammonia. Afterwards, the entire ion exchange process was repeated.

### 3.4. Scanning Electron Microscopy (SEM)

Small amounts of the calcined material were suspended in ethanol and applied to sample holders clad with adhesive aluminum foil or graphite pads. The conductivity of the dried samples was increased by sputtering with a gold platinum alloy. Images with a magnification up to 100,000 times were taken with a Hitachi SU 8000 microscope (Hitachi Europe, Düsseldorf, Germany).

### 3.5. Inductively Coupled Plasma Optical Emission Spectroscopy (ICP-OES)

The concentration of the elements Al, Si, Na, K, Ca, and Mg in the biosilica of different processing stages was determined via ICP-OES. Therefore, some milligram of the solid matter were digested in hydrofluoric acid (40%, Merck), hydrochloric acid (30%, Suprapur, Merck) and nitric acid (65%, Suprapur, Merck), 150 μL each. This mixture was heated up to 130 °C in 5 min via microwave in pressure vessels. After holding the temperature for 15 min at a pressure of 145 psi and 20 min cool-down, 1.5 mL boric acid (p.a., Fluka by Honeywell, Bucharest, Romania) was added. The sample was again treated in the microwave applying the same temperature program. Then, 0.15 mL nitric acid (65%) and 15 mL ultrapure water were added. 

The obtained clear liquid was diluted, depending on the analyte, and was subsequently analyzed with an Optima 7000DV spectrometer (Perkin Elmer, Rodgau, Germany). The measurement parameters were as follows: liquid flow 1.5 mL/min, plasma gas flow 16 L/min, auxiliary gas flow 1.6 L/min, nebulizer gas flow 0.7 L/min and 2-point background correction. The following spectral lines were utilized: 396.153 nm for Al, 212.412 nm for Si, 589.592 nm for Na, 766.490 nm for K, 317.933 nm for Ca, and 285.213 nm for Mg.

### 3.6. Nitrogen Physisorption

The surface area was characterized via BET (Brunauer, Emmett, Teller) method with a Quadrasorb SI Porosimeter (Quantachrome Instruments, Odelzhausen, Germany). Therefore, nitrogen was adsorbed at −196 °C. Prior to measurement, the samples were activated at 230 °C under dynamic vacuum (10^−3^ Pa) for 20 h to remove adsorbed water.

### 3.7. Temperature Programmed Desorption of Ammonia (NH_3_ TPD)

The acidity was examined with a BELCAT-Basic (Bel Japan Inc., Toyonaka, Japan). Initially, the dried samples were activated in a helium gas flow (50 mL/min) at 500 °C for 1 h. The substrates were then loaded in an ammonia gas flow (100%) of 50 mL/min for 30 min, and, hereafter, purged with a helium gas flow of 50 mL/min for 5 h to remove physisorbed ammonia. For desorption of the chemisorbed ammonia, the samples were heated with 10 °C/min to 450 °C under helium flow of 20 mL/min. Desorbed ammonia molecules were detected with a thermal conductivity detector (TCD).

### 3.8. Catalytic Tests

The following chemicals were employed for testing: 0.2 mL (~2 mmol) benzyl alcohol (99%, Alfa Aesar by Thermo Fisher, Karlsruhe, Germany), 8.8 mL (~100 mmol) benzene (Normapur, VWR, Darmstadt, Germany), used as reactant and solvent, and 30 µL (~0.1 mmol) tridecane (99%, VWR) as internal standard. The mixture was heated to 80 °C in a three-neck flask with reflux condenser, septum, and thermometer. 100 mg biosilica, beforehand activated at 230 °C via a Quantachrome Instruments FloVac Degasser, was added to start the reaction. Samples for GC-MS were taken through the septum after 0 h, 0.5 h, 1 h, 2 h, 4 h, 6 h and 8 h. A blank test without biosilica and a test with aluminum enriched, not ion-exchanged biosilica were carried out the same way. Calibrations for the reactants mentioned above, as well as for the expected products diphenylmethane (99%, abcr, Karlsruhe Deutschland) and dibenzyl ether (purum, Fluka) were carried out.

### 3.9. Gas Chromatography Coupled to Mass Spectrometry (GC-MS)

The catalytic reaction was monitored using an Agilent 6890N Network GS System with a 5973N Mass Selective Detector (Agilent Technologies, Waldbronn, Germany). The parameters of the gas chromatograph were as follows: split/splitless injection at 260 °C, carrier gas helium with a gas flow of 1 mL/min and capillary column Macherey-Nagel (Düren, Germany) Optima-1, with 100% polysiloxane film (0.2 µm thickness) and 12.5 m length, as well as 0.2 mm inner diameter. The transfer line temperature was 280 °C.

The parameters of the mass spectrometer were configured as: electron impact ionization at 230 °C with 70 eV, quadrupole mass filter at 150 °C with a range from *m*/*z* 42 to 500, detection by a secondary electron multiplier after 1 min solvent delay.

The following method was developed to separate reactant and products: holding a temperature of 50 °C for 3 min, then heating with 25 °C/min to 110 °C, afterwards heating with 15 °C/min to 155 °C, and subsequently with 25 °C/min to 300 °C. Chromatograms were normalized to the internal standard.

## 4. Conclusions

The small, robust, and fast growing diatom *T.p.* was cultured under an elevated aluminum concentration. No perturbations occurred except for a prolonged growth phase. After harvesting, cell wall extraction and cleaning, the frustules were also ion exchanged with ammonium chloride. The obtained materials were characterized with SEM, ICP-OES, N_2_ physisorption, and NH_3_ TPD.

SEM showed the common morphology of *T.p.* in both the aluminum enriched and the in vitro modified material. That means the characteristic frustule structure was preserved. The aluminum enriched material exhibits a specific surface area of 55 m^2^/g. Elemental analysis revealed that the relative amount of aluminum increased from a molar Al:Si ratio of 1:600 to a ratio of 1:50. The concentration of sodium increased simultaneously. As expected, the concentration of all the analyzed alkali and alkaline earth metals, i.e., Na, K, Ca, and Mg, declined during the ion exchange and subsequent calcination. By introducing acidic protons in this modification step, the acidity was more than doubled from 149 to 320 µmol NH_3_/g. 

The aluminum enriched and ion exchanged biosilica was proven to be catalytically active for the acid-catalyzed reaction of benzene and benzyl alcohol to diphenylmethane and dibenzyl ether. These observations represent a first step in the implementation of diatom silica as acid catalyst material. When considering the various possibilities to create biosilica with higher catalytic activity, modified diatom biosilica indeed forms a promising source for “green” catalyst synthesis. The ecofriendly nature of diatoms and their inexpensive, scalable cultivation could make them particularly interesting for novel industrial production lines.

## Figures and Tables

**Table 1:** Elemental composition of the calcined biosilica as determined by Inductively Coupled Plasma Optical Emission Spectroscopy (ICP-OES).

	*T.p.* Reference		*T.p.* + Al		*T.p.* + Al + Ion Exchange	
Element	$\frac{n(x)}{n(Si)}\times 10^{3}$	*n*(x):*n*(Si)	$\frac{n(x)}{n(Si)}\times 10^{3}$	*n*(x):*n*(Si)	$\frac{n(x)}{n(Si)}\times 10^{3}$	*n*(x):*n*(Si)
Al	1.66	1:603	21.4	1:47	16.3	1:61
Na	39.1	1:26	58.2	1:17	7.35	1:136
K	0.307	1:3250	5.29	1:189	0.239	1:4190
Ca	0.451	1:2220	1.40	1:715	0.614	1:1630
Mg	2.58	1:387	0.828	1:1208	0.503	1:1990

## Supplementary Materials

Supplementary materials are available online.
