# Supplementary material for: Are Diatoms “Green” Aluminosilicate Synthesis Microreactors for Future Catalyst Production?
Source: Molecules. 2017 Dec 16;22(12):2232. doi: 10.3390/molecules22122232 (PMC6149991; doi:10.3390/molecules22122232)
Supplement: Supplementary file 1 [file molecules-22-02232-s001.pdf]

Supplementary Material

Table S1. Composition of the Culture Medium ASW

Stock solution	Substance	β_{ASW} in g · l^{-1}	c_{ASW} in mmol · l^{-1}
**B1**	NaCl	$2.08 \cdot 10$	$3.55 \cdot 10^{2}$
	Na_2SO_4	3.48	$2.45 \cdot 10$
	KCl	$5.87 \cdot 10^{-1}$	7.87
	$NaHCO_3$	$1.70 \cdot 10^{-1}$	2.02
	KBr	$8.45 \cdot 10^{-2}$	$7.10 \cdot 10^{-1}$
	H_3BO_3	$2.20 \cdot 10^{-2}$	$3.56 \cdot 10^{-1}$
	NaF	$2.70 \cdot 10^{-3}$	$6.43 \cdot 10^{-2}$
**B2**	$MgCl_2 \cdot 6\ H_2O$	9.40	$4.62 \cdot 10$
	$CaCl_2 \cdot 2\ H_2O$	1.32	8.95
	$SrCl_2 \cdot 6\ H_2O$	$2.14 \cdot 10^{-2}$	$8.03 \cdot 10^{-2}$
**Z1**	$Na_2SiO_3 \cdot 9\ H_2O$	$3.00 \cdot 10^{-2}$	$1.06 \cdot 10^{-1}$
**Z2**	$NaNO_3$	$4.67 \cdot 10^{-2}$	$5.49 \cdot 10^{-1}$
**Z3**	Glycerophosphate disodium salt hydrate	$6.67 \cdot 10^{-3}$	$3.09 \cdot 10^{-2}$
**Z4***	Titriplex III	$3.64 \cdot 10^{-3}$	$9.78 \cdot 10^{-3}$
	$FeCl_3 \cdot 6\ H_2O$	$1.77 \cdot 10^{-3}$	$6.55 \cdot 10^{-3}$
**Z5**	$MnCl_2 \cdot 4\ H_2O$	$4.78 \cdot 10^{-4}$	$2.42 \cdot 10^{-3}$
	$ZnCl_2$	$3.46 \cdot 10^{-5}$	$2.54 \cdot 10^{-4}$
	$CoSO_4 \cdot 7\ H_2O$	$1.60 \cdot 10^{-5}$	$5.69 \cdot 10^{-5}$
	$Na_2MoO_4 \cdot 2\ H_2O$	$1.26 \cdot 10^{-4}$	$5.21 \cdot 10^{-4}$
	$C_{10}H_{14}N_2Na_2O_8 \cdot 2\ H_2O$ (Titriplex III. EDTA)	$1.89 \cdot 10^{-3}$	$5.08 \cdot 10^{-3}$
**Z6**	$Na_2SeO_3 \cdot 5\ H_2O$	$2.63 \cdot 10^{-6}$	$1.00 \cdot 10^{-5}$
**Z7**	$CuCl_2$	$1.34 \cdot 10^{-4}$	$9.97 \cdot 10^{-4}$
**V1**	Thiamine · HCl	$2.24 \cdot 10^{-4}$	$6.64 \cdot 10^{-4}$
**V2**	Cobalamin	$4.00 \cdot 10^{-6}$	$2.95 \cdot 10^{-6}$
**V3**	Biotin	$2.00 \cdot 10^{-6}$	$8.19 \cdot 10^{-6}$
